# Why Hospital Patients Complain

**Published:** 1896-04-25

**Authors:** 


### HOME HOSPITALS' ASSOCIATION.

### WHY HOSPITAL PATIENTS COMPLAIN.

Mr. W. H. Bennett, F.R.C.S., at the annual meeting of the Home Hospitals Association, stated that the attendance, comfort, food, nursing, and every accessory connected with Fitzroy House were so excellent that patients felt their residence there was equivalent to residence at home. He had had patients in the Home Hospital from its earliest days, and only one patient had ever complained of anything. This gentleman was very particular about his wine, and prided himself upon the excellence of his cellar. He requested that the rules might be suspended in his case, and that he might bring his own wine with him, which he accordingly did. In a little while the patient complained that the wine was not fit to drink! We commend this story to our colleagues on the lay press, as showing that all complaints by hospital patients should be received with a due amount of scepticism. The truth is that the physical condition of the patient during illness often causes a general distaste for food and nourishment of all kinds, a point which is often overlooked. No more forcible instance of this fact can be adduced than that of a connoisseur in wines, who, having spent many years of his life in completing his cellar, found during the period of his treatment by an eminent surgeon that none of his wines were fit to drink!

